# Natural products as reservoirs of novel therapeutic agents

**DOI:** 10.17179/excli2018-1174

**Published:** 2018-05-04

**Authors:** Sadaf Mushtaq, Bilal Haider Abbasi, Bushra Uzair, Rashda Abbasi

**Affiliations:** 1 Department of Biotechnology, Faculty of Biological Sciences, Quaid-i-Azam University Islamabad-45320, Pakistan; 2 EA2106 Biomolécules et Biotechnologies Végétales, Université de Tours, 37200 Tours, France; 3 Department of Bioinformatics & Biotechnology, Faculty of Basic & Applied Sciences, International Islamic University, Sector H-8, Islamabad, Pakistan; 4 Institute of Biomedical & Genetic Engineering (IBGE), Sector G-9/1, Islamabad, Pakistan

**Keywords:** bioactive, secondary metabolites, plants, animals, marine organisms, microbes

## Abstract

Since ancient times, natural products from plants, animals, microbial and marine sources have been exploited for treatment of several diseases. The knowledge of our ancestors is the base of modern drug discovery process. However, due to the presence of extensive biodiversity in natural sources, the percentage of secondary metabolites screened for bioactivity is low. This review aims to provide a brief overview of historically significant natural therapeutic agents along with some current potential drug candidates. It will also provide an insight into pros and cons of natural product discovery and how development of recent approaches has answered the challenges associated with it.

## Introduction

The word “Bioactive” is a unification of two terms from the Greek language: “bios” means “life” and “activus” means energetic or active. Hence, “Bioactive” is an alternate word for “Biologically Active”. A chemical entity having some biological activity in living organisms is considered bioactive. This biological activity may include a reaction or induction of a response. The effect can be favourable or unfavourable depending on the active agent, the dose used and its biological availability (Guaadaoui et al., 2014[52]). 

The extracts prepared from natural sources contain structurally diverse compounds called secondary metabolites. Metabolism is divided into two main categories in living organisms: Primary and Secondary metabolism. Primary metabolites include biological molecules i.e., nucleic acids, fats, carbohydrates and proteins, essential for the survival and well-being of the organism. Secondary metabolism involves biosynthesis of secondary metabolites (natural products). These compounds are exclusive to an organism and play role in defence mechanisms against predators (Dias et al., 2012[40]). The major difference between primary and secondary metabolites lies in their biological effects. Primary metabolites exert their biological effect on cell or body of the organism. Whereas, secondary metabolites exert biological effect within the organism and also on other organisms (Jabeen et al., 2014[59]). These products can be harvested directly from microbial fermentation, tissue samples of plants and marine organisms or synthesized on an industrial scale (Lahlou, 2013[68]). According to an estimate, more than 300,000 secondary metabolites exist in nature (Gurnani et al., 2014[54]).

The biological processes responsible for the production of secondary metabolites include photosynthesis, glycolysis and Kreb cycle. These biological processes result in the biosynthesis of “Shunt metabolites” or intermediates which proceed towards manufacturing of secondary metabolites via alternate biosynthetic routes responsible for metabolite diversity in living organisms. Some examples of intermediates comprise of shikimic acid, 1-deoxyxylulose-5-phosphate, acetyl coenzyme A and mevalonic acid. The modification in biosynthetic routes of intermediates may be due to environmental changes, viral, chemical or radiation exposure (Dias et al., 2012[40]).

**Figure 1:**
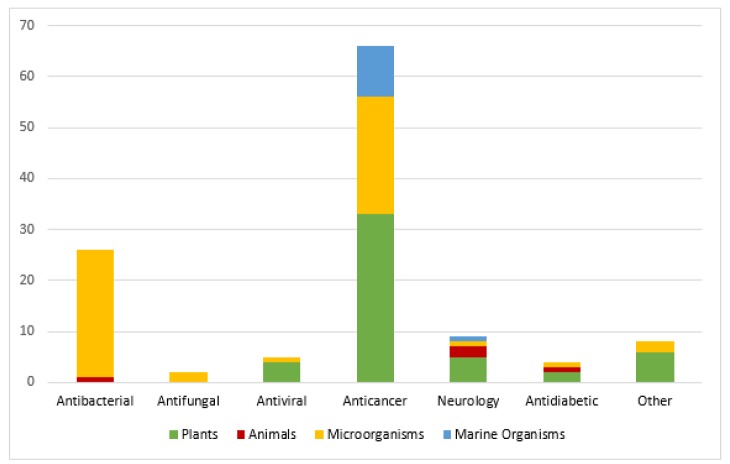
Classes of natural drugs under clinical trials and their sources (Brahmachari, 2012)

Nature is a valuable reservoir of novel bioactive entities. According to an estimate, about 50 % of the medications validated from 1981-2010 have natural origins (i.e., 28 % semi-synthetic, 17 % mimics of natural compounds and 5 % natural entities). Similarly, in the discipline of cancer therapeutics, natural products hold paramount potential. From 1940-2010, 175 anti-cancer drugs have been developed. Out of these, 48.6 % drugs have either natural origins or they are derived from natural products (Gurnani et al., 2014[54]). Many natural drugs are reported to be in various phases of clinical trials as well (Figure 1(Fig. 1), Reference in Figure 1: Brahmachari, 2012[29]). 

Despite the advancements in modern techniques such as Nuclear Magnetic Resonance (NMR), X-ray crystallography and alternate drug discovery methods including rational drug designing and combinatorial chemistry, lead compounds progressing towards clinical trials are still a deficit. Similarly, the emergence of multidrug-resistant microbes has increased the urge to search for new therapeutic lead compounds. Natural compounds are still a source of compelling leads in the fields of oncology, metabolic disorders and immunosuppression due to increased chemical diversity as compared to synthetic compounds (Butler, 2005[30]; Monciardini et al., 2014[79]). 

### History of natural products as therapeutics

History of medication is as old as human civilization (Lahlou, 2013[68]). Use of natural products as the medication has been proven by ancient written documents originated from India, North Africa and China (Phillipson, 2001[156]). The earliest acknowledged medical document was a Sumerian clay tablet with written remedies for numerous ailments, dating back to 4000 years. Examples of such remedies include the use of garlic for circulatory and heart disorders and Mandrake for pain relief (Ming et al., 2003[78]). According to Chinese herb guide, the use of herbal medicine dates back to 2000 BC. Chinese drug encyclopedia, “Pen-ts'as kang mu”, has documented about 1898 herbal drugs and 8160 prescriptions. “Ebers Papyrus”, an Egyptian document, has reported 1000 different herbs and formulations as medicines. Similarly, a Greek document, “De Materia Medica” which dates back to AD 100, is also based on the use of herbs in medicine (Brahmachari, 2012[29]). Evidence of plant-based medicine in India has been found in 1000 BC old collection of Ayurvedic hymns which reported the use of 1000 different herbs as medication (Gurnani et al., 2014[54]).

Today, the modern medicine has been developed on the basis of scientific knowledge and observational attempts of scientists. But, this knowledge has been established majorly from ancient knowledge of our ancestors (Brahmachari, 2012[29]).

### Advantages of natural entities in drug discovery

- Natural entities exhibit tremendous chemical and structural diversity. They have drug-likeness and biological friendliness features with high molecular weights, more oxygen atoms and complex structures enabling them to exhibit advanced binding characteristics. Structural diversity also allows researchers to create new chemical entities by using computational molecular modelling techniques.
- Synthesis of natural products is a result of enzymatic interactions. Therefore, their biological activity involves protein binding, making them effective drug candidates.
- Secondary metabolites are a product of evolutionary pressure responsible for their novelty. They are more prone to have bioactivity compared to synthetic compounds (Lahlou, 2013[68]).

### Natural drug development process

The process of drug development from natural products is based on following two strategies:

- Assessment of biological activities of pure compounds (chemically driven method).
- Bioassay-guided process which involves working with crude extracts (biologically driven method).

Academic-based research objectives have shifted to the biologically driven method. Whereas, the chemically driven method involves following steps:

- Natural product identification.
- Initial screening of the crude extract.
- Extraction of secondary metabolite.
- Structure elucidation of secondary metabolites by spectroscopic techniques namely High Profile Liquid Chromatography (HPLC) and 2D Nuclear Magnetic Resonance (NMR).
- Assessment of biological activities of purified compounds *in vitro* and *in vivo*.
- Preclinical studies to understand pharmacokinetics and mechanism of action of the selected bioactive compound.
- Isolated products which are proven more effective than existing drugs are selected and various economical methods are designed for bulk production.
- Finally, in clinical trials, selected drugs are tested for efficacy and safety on human volunteers.
- After approval, drugs are introduced in the market. In case of poor bioavailability, chemical modification or derivatization is done to increase efficacy (Gurnani et al., 2014[54]).

## Sources of Natural Products

Natural products can be obtained from four main sources i.e., plants, animals, marine organisms and microorganisms (Jabeen et al., 2014[59]).

### Natural products from plants

Plants species have been reported to have medicinal use since ancient times with better patient tolerance and acceptance. Evolution has resulted in better adaptation and production of structurally diverse secondary metabolites. According to World Health Organization (WHO), 80 % of the world's population is committed to folk medicine derived from plants for primary health care. Knowledge related to traditional medicine has been a major factor for investigation of medicinal plants and production of pharmaceuticals. About 80 % of plant-based medicines are in accordance with their original ethnopharmacological function (Dias et al., 2012[40]).

The plant kingdom comprises of 300,000-400,000 higher species. According to an estimate, only 5-15 % of terrestrial plant species have been investigated pharmacologically. 10,000-15,000 of world's plant species have been reported as medicinal plants and about 150-200 species have been integrated into western medicine. About 25 % of all medicines today have plants origins (Gurnani et al., 2014[54]).

**Figure 2:**
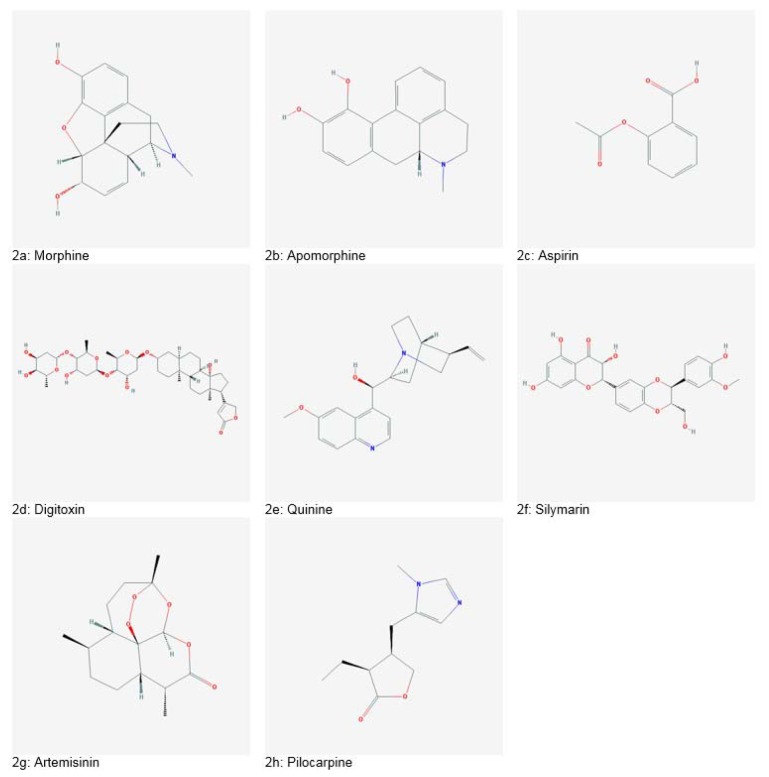
Historically significant plant-derived drugs (Source: National Centre for Biotechnology Information. PubChem Compound Database: CIDs from 2a-2h = 5288826, 6005, 2244, 441207, 3034034, 1548994, 68827 and 5910)

The first natural and pure plant-derived medicine was morphine (Figure 2a(Fig. 2)) isolated from *Papaver somniferum* in 1803, commercialized in 1826 by Merck. In the 1870s, morphine in crude form was boiled in acetic anhydride, producing heroine which was readily converted into codeine, used as a painkiller. Apomorphine (Figure 2b(Fig. 2)), derived from morphine acts as an agonist of Dopamine receptor D1 and D2. It has been utilized for the treatment of Parkinson's disease. Another famous drug is aspirin (Figure 2c(Fig. 2)), an anti-inflammatory agent isolated from the bark of *Salix alba, *introduced by Bayer in 1899. Digitoxin (Figure 2d(Fig. 2)), a potent cardiotonic glycoside was isolated from *Digitalis purpurea*. It is used for the treatment of congestive heart failure. An antimalarial drug named as Quinine (Figure 2e(Fig. 2)), was obtained from *Cinchona succirubra *bark. It got approval by Food and Drug Administration (FDA) in 2004. Quinine is a drug of choice for numerous ailments including malaria, dyspepsia, fever, mouth and throat diseases, and cancer (Deleu et al., 2004[38]; Dias et al., 2012[40]). Silymarin (Figure 2f(Fig. 2)), a drug for treatment of liver disorders was isolated from *Silybum marianum*. Silymarin is also reported to exhibit anticancer, antiviral, anti-fibrotic, antioxidant, immunomodulatory and anti-inflammatory potential (Abbasi et al., 2016[2]; Ahmad et al., 2013[8][9]; Khan et al., 2013[63], 2014[64]; Mahmood et al., 2010[74]). Artemisinin (Figure 2g(Fig. 2)) produced from *Artemisia annua* is a treatment option for multidrug-resistant malaria. Traditionally, artemisinin has also been used as an antiseptic, antidiabetic, antispasmodic, digestive, choleretic, diuretic, trematocidal, anti-helminthic and depurative agent (Ali et al., 2013[16], 2015[15], 2016[13], 2017[14]; Tariq et al., 2014[173]). Another important drug, pilocarpine (Figure 2h(Fig. 2)) was isolated from *Pilocarpus jaborandi*. It has been used for treating glaucoma and management of xerostomia and Sjogren's syndrome (Dias et al., 2012;[40] Veeresham, 2012[177]). Table 1(Tab. 1) (References in Table 1: Abbasi et al., 2007[6], 2009[4], 2010[1], 2012[5], 2017[3]; Ahmad et al., 2010[10], 2016[7]; Alzohairy, 2016[17]; Anjum et al., 2017[19][20]; Biswas et al., 2002[27]; Chen and Huang, 2005[33]; Coman et al., 2012[35]; Du et al., 2012[41]; Gamboa-Gomez et al., 2015[47]; Ghosh and Sil, 2013[48]; Gomez-Flores et al., 2016[49]; Jurenka, 2009[60]; Lee and Scagel, 2013[69]; Narinderpal et al., 2013[81]; Nino et al., 2009[147]; Nugroho et al., 2012[150]; Quintanilla-Licea et al., 2014[160]; Recio et al., 2012[162]; Siddiqui et al., 2014[168]; Singh et al., 2011[169]; Thakur et al., 2016[174]; Zaka et al., 2017[184]) enlists some significant plant-based bioactive entities.

#### Plant based anticancer drugs

Worldwide, cancer has been identified as a significant cause of deaths (Bhanot et al., 2011[26]). Globocan report stated 14.1 million cancer cases in 2012 with 8.2 million deaths worldwide. The most common were cancers of lung, breast and colon. Cancer burden is expected to increase to over 20 million new cases expected in 2025 (Ferlay et al., 2015[43]). The primary objective of the cancer research is to search for novel, less toxic and more potent drug leads which act specifically on cancer cells or search for targeted drug delivery method (Isnard-Bagnis et al., 2005[58]; Phonnok et al., 2010[157]).

About 1000 different plant species have anticancer potential. Plant-based drug discovery process is based on three perspectives:

- Determination of bioactivity of the compound including mode of action, its isolation from extract and characterization.
- Rational drug designing which includes analogue synthesis and structural modifications (Priya et al., 2015[158]).

**Figure 3:**
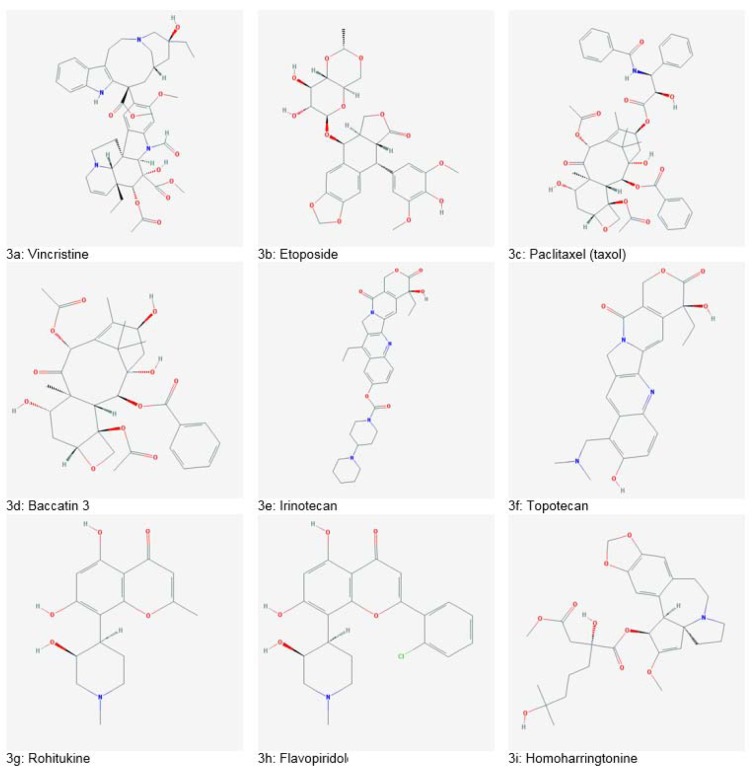
Plant-derived anticancer drugs (Source: National Center for Biotechnology Information, PubChem Compound Database: CIDs from 3a-3i = 5978, 36462, 36314, 65366, 60838, 60700, 13422573, 5287969 and 285033)

Important plant-based anticancer drugs include Vincristine (Figure 3a(Fig. 3)), Vinblastine (isolated from Madagascan periwinkle, *Catharanthus roseus*) and Etoposide (Figure 3b(Fig. 3)) isolated from mandrake plant *Podophyllum peltatum*, which are antimitotic drugs (Bhanot et al., 2011[26]; Noble, 1990[149]). Paclitaxel/ taxol (Figure 3c(Fig. 3)), produced from Pacific yew (*Taxus brevifolia)* bark is commonly used for treating breast cancers. It promotes tubulin stabilization leading towards apoptosis. Baccatin 3 (Figure 3d(Fig. 3)), a structural analogue of taxol, is isolated from needles of *T. brevifolia* and converted into taxol efficiently on a commercial scale (Dias et al., 2012[40]; Wilson and Jordan, 1995[179]). Irinotecan (Figure 3e(Fig. 3)) and topotecan (Figure 3f(Fig. 3)) isolated from bark and wood of *Camptotheca accuminata *have cytotoxic effects on colorectal and ovarian cancers by topoisomerase inhibiting mechanism. Rohitukine (Figure 3g(Fig. 3)), an alkaloid, was obtained from leaves and stems of *Dysoxylum binectariferum*. Flavopiridol (Figure 3h(Fig. 3)), a flavone synthesized from rohitukine, was the first drug using cyclin-dependent kinase inhibition mechanism. Homoharringtonine (Figure 3i(Fig. 3)), isolated from *Cephalotaxus harringtonia*, has potency against various leukemias. It works by blocking protein synthesis and cell cycle progression (Bhanot et al., 2011[26]). *Euphorbia peplus* is a producer of Ingenol 3-angelate (I3A) which has shown potential against various skin cancers and resulted in excellent skin cosmesis in mice. I3A acts as a ligand of protein kinase C (PKC) (Kedei et al., 2004[62]; Ogbourne et al., 2004[151]). Genus *Fagonia* comprises of various species with health-promoting tendencies. Many species have been reported to accumulate several bioactive compounds such as flavonoids, phenols, alkaloids, tannins, glycolides, saponins, coumarins, sterols and terpenoids. Among these, *Fagonia indica* has gained interest as a potent anticancer agent. It has been marketed as “virgin's mantle tea” for breast cancer. The extracts of *Fagonia indica* have also shown antimicrobial, antidiabetic, antioxidant, hepatoprotective, analgesic and anti-inflammatory potential (Khan et al., 2016[66], 2017[65]). Some medicinally important plants and their anticancer compounds are enlisted in Table 2(Tab. 2) (References in Table 2: Amin et al., 2009[18]; Greenwell and Rahman, 2015[51]; Nirmala et al., 2011[148]; Priya et al., 2015[158]; Sain and Sharma, 2013[164]; Sisodiya, 2013[171]; Weaver, 2014[178]).

### Natural products from animals

Animals based therapy called zoo-therapy has been used in almost every culture of the world. Sources of animal-based therapeutic compounds include body parts and metabolic produce (including excrements and corporal secretions).

**Figure 4:**
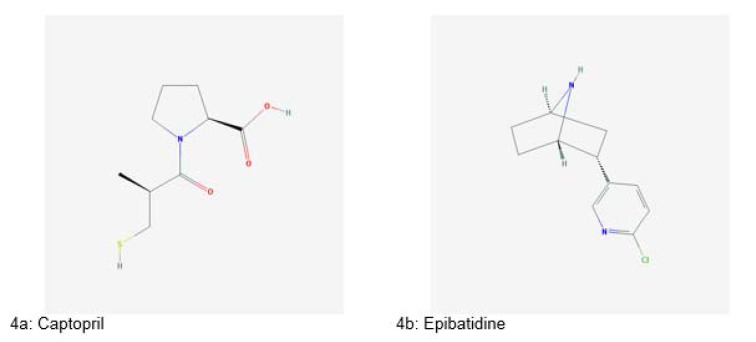
Significant drugs derived from animals (Source: National Center for Biotechnology Information, PubChem Compound Database: CIDs from 4a-4b = 44093 and 3073763)

The exploitation of animals as sources of therapeutics is based on indigenous knowledge of our ancestors. For example, ants of *Pseudomyrmex* genus were squashed and used in a toothache. They were also left to bite joints to get relief from joint pain. Body parts of rattlesnake were used for the treatment of boils and bronchitis (Costa-Neto, 2005[36]). Snake venoms were used for the treatment of arthritis and gastric disorders from the 7^th^ century. The venom of cobra snake has been used for treating various ailments such as polio, pain, multiple sclerosis and asthma since the 1930s. Captopril (Figure 4a(Fig. 4)), an antihypertensive drug, was derived from the venom of Brazilian viper *Bothrops jararaca* in 1970s (King, 2013[67]). 

Insects have also played a significant part in the development of modern medicine. Chitosan, an insect compound isolated from chitin has anticoagulant properties. It has been reported to lower serum cholesterol levels and facilitates tissue repair. Traditionally, leeches (*Hirudo medicinalis*) were used to treat abnormal swellings, abscess, piles, poisonous bites, and rheumatoid arthritis etc. Salivary glands of leech contain a vasodilator, an anticoagulant, a local anaesthetic, and antibiotic compounds. Studies have shown that leech anticoagulant compound is more effective than heparin. It is smaller in size and penetrates the clot more effectively and inhibits fibrin formation (Costa-Neto, 2005[36]).

Another important therapeutic compound isolated from an animal source is epibatidine (Figure 4b(Fig. 4)), a potent analgesic agent, isolated from a frog *Epipedobates anthonyi*. It is ten times more effective than morphine (Fitch et al., 2010[45]). 

### Natural products from marine organisms

About 70 % of Earth's area is covered with oceans. The biodiversity in marine ecosystems is greater than that of tropical rainforests which has resulted in structurally diverse secondary metabolites produced by marine microfauna and microflora of which, very few have been studied for bioactivities (Haefner, 2003[55]).

**Figure 5:**
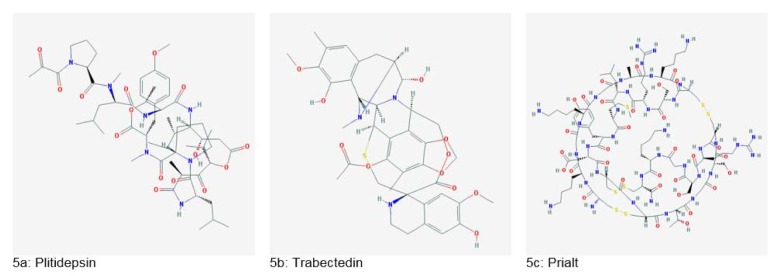
Important marine-derived drugs (Source: National Center for Biotechnology Information, PubChem Compound Database: CIDs from 5a-5c = 9812534, 108150 and 16135415)

A depsipeptide, Plitidepsin (Figure 5a(Fig. 5)), produced from *Aplidium albicans*, has shown a potential anticancer effect against several cancers. This drug is also in Phase 2 clinical trials. Ecteinascidin, also known as Trabectedin (Figure 5b(Fig. 5)) was isolated from *Ecteinascidia turbinata.* It was the first anticancer agent from the marine source to gain approval by European Union (Dias et al., 2012[40]). Prialt (Figure 5c(Fig. 5)), a calcium channel blocker peptide was introduced in 2004 and used for the management of chronic pain. Also, ShK peptide from sea anemone has shown potential for multiple sclerosis and other autoimmune diseases (King, 2013[67]). 

#### Marine sponges

Marine sponges are immobile in nature. Sponges are said to be the first multicellular organisms and they have diversified very little in about 500 million years (Dias et al., 2012[40]). 

**Figure 6:**
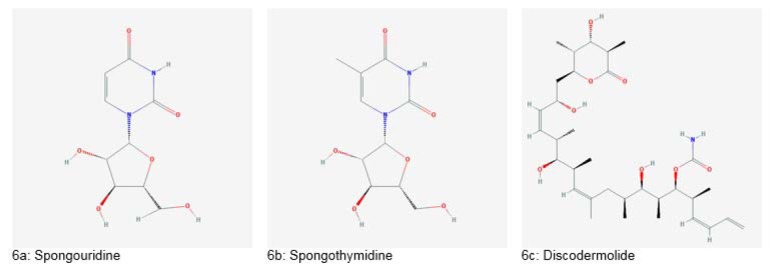
Important drugs derived from marine sponges (Source: National Center for Biotechnology Information, PubChem Compound Database: CIDs from 6a-6c = 18323, 65049 and 643668)

The search for marine bioactive compounds began in the 1950s after the isolation of “Spongothymidine” and “Spongouridine” from *Crytotheca crypta* (Figure 6a and 6b(Fig. 6)). These nucleotides showed great efficacy as antiviral and anticancer drugs (Haefner, 2003[55]). Further exploration of the marine environment became possible after the introduction of Self-Contained Underwater Breathing Apparatus (SCUBA) in the 1970s and remotely operated vehicles (ROVs) in 1990s (Dias et al., 2012[40]).

Another example is “Discodermolide” produced by *Discodermia dissolute *(Figure 6c(Fig. 6)). This drug has stabilizing action just like taxol and has shown cytotoxic effects against drug-resistant tumors and taxol-resistant tumors in clinical evaluation. Also, combination therapy of discodermolide and taxol showed better tumor reduction *in vivo* (Huang et al., 2006[56]). 

#### Marine algae

About 30,000 species of algae are inhabitants of marine ecosystems worldwide. Algae have been used as a source of food, medicine and fertilizers by various ethnic groups. Ancient knowledge and ample availability of algal species is the basis of modern algal research. A famous class of compounds isolated from algal species is terpenoids and terpenoid like structures e.g., phenazine derivatives, sterols, brominated oxygen and nitrogen heterocycles, amino acids and amines (Dias et al., 2012[40]; Torres et al., 2014[176]). 

Numerous bioactive entities isolated from algae have anti-oxidative, anti-inflammatory and anticancer characteristics. Extracts of red algae, *Callophyllis japonica* and *Gracilaria tenuistipitata* have demonstrated anti-oxidative potential. *C. japonica* enhanced activation of antioxidant enzymes and inhibited hydrogen peroxide (H_2_O_2_) induced apoptosis. Similarly, extracts of *G. tenuistipitata* helped in enhanced recovery of human lung carcinoma cells (H1299) from DNA damage (Lee et al., 2013[70]). *Ulva lactuca* and *S. wightii* have been reported to produce flavonoids which play role in antioxidant defense system of the body (Meenakshi et al., 2009[77]). *Ulva reticulata* extracts demonstrated anti-hepatotoxic properties *in* *vivo* (Rao et al., 2004[161]).

**Figure 7:**
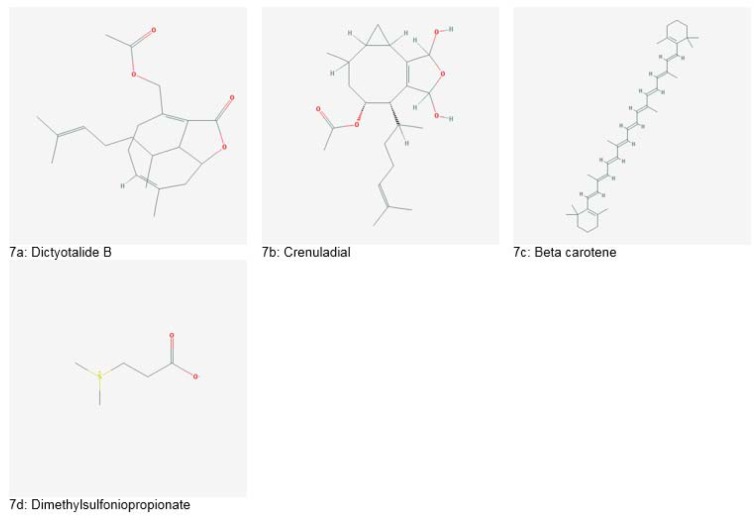
Important bioactive entities derived from marine algae (Source: National Center for Biotechnology Information, PubChem Compound Database: CIDs from 7a-7d = 6443354, 365667, 5280489 and 23736)

Several antitumor compounds such as, diterpenes, dictyolides A, B (Figure 7a(Fig. 7)), 4-acetoxydictylolactone and nordictyolide have been isolated from a brown alga, *Dictyota dichotoma*. Similarly, another brown alga, *Dilophus ligatus*, is a producer of crenuladial (Figure 7b(Fig. 7)). This compound has shown potent antimicrobial activity against *Micrococcus luteus, Aeromonas hydrophyla* and *Staphylococcus aureus* (Dias et al., 2012[40]).

Two species, *G. verrucosa* and *G. textorii* have been reported to have anti-inflammatory potential. Another example is *Porphyridium spp*., from which a retrovirus inhibiting polysaccharide was isolated. Methanolic extract of *Neorhodomela* *aculeate* showed suppression of reactive oxygen species (ROS) production, lipid peroxidation and nitric oxide synthase. Beta carotene (Figure 7c(Fig. 7)), obtained from a green algae *Dunaliella bardawil*, exhibited protective effects against inflamed bowel in mice models. Also, phlorotannins isolated from species of brown algae: *Eisenia bicyclis*, *Ecklonia kurome* and *Ecklonia cava* have shown antioxidant potential (Lee et al., 2013[70]). A blue green alga, *Anabaena spp*. also showed radical scavenging activity (Pant et al., 2011[154]).

Some species of marine algae possess anticancer potential. Extracts of *Gracilaria tenuistipitata* were reported to have growth inhibitory effects on Ca9-22 oral cancer cells by causing oxidative stress in cancer cells (Yeh et al., 2012[182]). Another species, *Plocamium telfairiae* induced apoptosis in HT-29 colon cancer cells. Green algae produce a tertiary sulfonium metabolite called Dimethylsulfoniopropionate (Figure 7d(Fig. 7)), which has significant anticancer potential against Ehrlich ascites carcinoma *in vivo*. Similarly, heterofucans, isolated from *Sargassum filipendula* possess anticancer effects against cervical, prostate and liver cancer cells (Lee et al., 2013[70]).

#### Marine bacteria

Marine microorganisms are valuable sources of antibacterial, antifungal and antiviral compounds. But the process of marine compounds incorporation in therapeutics is slow because information regarding ethnomedical history is lacking. Today, various pharmaceutical companies have developed programs for isolation and characterization of marine bioactive compounds (Debnath et al., 2007[37]).

Among marine bacteria, *Actinomycetes* are the largest contributors of novel bioactive secondary metabolites accounting for 45 % of total microbial metabolites (75 % from *Streptomyces *and 25 % from rare *Actinomycetes*). A huge variety of antibiotics such as anthracyclines, aminoglycosides, glycopeptides, beta-lactams, macrolides, polyketides, actinomycins and tetracyclines have been isolated from *Actinomycetes* (Subramani and Aalbersberg, 2012[172]).

**Figure 8:**
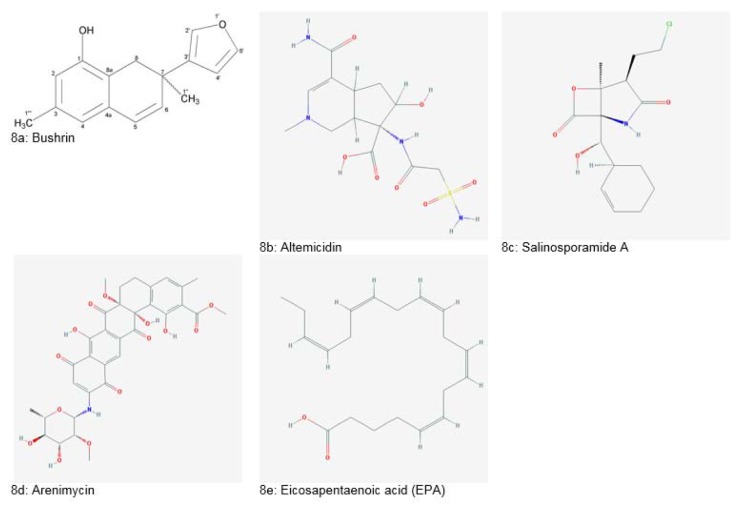
Natural products derived from marine bacteria (Source 8a: Ahmed et al., 2008; National Center for Biotechnology Information, PubChem Compound Database: CIDs from 8b-8e = 6711682, 11347535, 46216699 and 446284)

*Nocardia spp.* is a producer of a group of novel maytansinoid antibiotics called “Ansamitocins”. Ansamitocins exhibited significant antitumor and antifungal activities. *Vibrio marinus*, an isolate of Baltic Sea, it was reported to produce antiviral compounds effective against enteroviruses. A novel antibiotic named “Bushrin” (Figure 8a(Fig. 8), Reference in Figure 8: Ahmed et al., 2008[11]) was extracted from *Pseudomonas stutzeri*, an isolate of ribbonfish of Baluchistan coast, Pakistan. The compound showed high efficacy against several gram-positive and negative bacteria (Ahmed et al., 2008[11]). Another species, *Pseudomonas bromoutilis*, was reported to produce a novel pyrrole antibiotic which showed significant inhibition of gram-positive bacteria. Antibiotic MC21-A was isolated from *Pseudoalteromonas phenolica* which exhibited bactericidal effect (Debnath et al., 2007[37]).

Altemicidin (Figure 8b(Fig. 8)), isolated from *Streptomyces sioyaensis* had both antitumor and acaricidal activity. It also showed inhibition against *Xanthomonas *strains (Debnath et al., 2007[37]). Salinosporamide A (Figure 8c(Fig. 8)), obtained from *Salinispora tropica* was reported to induce apoptosis in multiple myeloma cells (Subramani and Aalbersberg, 2012[172]). Another novel entity, lodopyridone was extracted from genus *Saccharomonospora*. This alkaloid showed cytotoxicity in human colon adenocarcinoma (HCT-116) cells (Maloney et al., 2009[75]). 

Isolation of Arenimycin (Figure 8d(Fig. 8)) antibiotic from *Salinispora arenicola* was reported by Asolkar et al. (2010[21]). It showed a potent antimicrobial effect against rifampin and methicillin-resistant *Enterococcus feacalis*, *Enterococcus faecium* and *Staphylococcus aureus*. Arenimycin also induced cytotoxicity in human adenocarcinoma cells.

Some bacteria such as *Vibrio vulnificus*, produce specific proteins called “Bacteriocins” which are bactericidal towards closely related species and are also used for preserving seafood (Shehane and Sizemore, 2002[167]).

Another important discovery was the isolation of bacterium SCRC-2738 from the intestine of Pacific mackerel. This bacterium produced eicosapentaenoic acid (Figure 8e(Fig. 8)) which is used as a preventive and curative agent for thrombosis atherosclerosis (Debnath et al., 2007[37]). 

### Natural products from microbes

Bacteria and fungi are inhabitants of diverse habitats worldwide. Due to which, they have evolved to cope with adverse conditions. The structurally diverse secondary metabolites produced by them possess biological activities such as antibiotic, anti-inflammatory, anticancer and antidiabetic activities (Singla et al., 2014[170]). 

According to an estimate, only 5 % of world's fungal and 0.1 % of bacterial species are known to man and a very little fraction has been screened for their bioactivity (Thomas et al., 2011[175]). Today, more than 1 million natural compounds have been isolated from which 50-60 % have plant and 5 % have microbial origins. Among the natural products, 20-25 % has been classified as biologically active. From these, 10 % have been isolated from microbes, 45 % from *Actinomycetes*, 38 % from fungi, and 17 % from unicellular bacteria (Demain and Sanchez, 2009[39]).

The important characteristics of microbial bioactive compounds include their specific microbial origin, environmental interactions and unique chemical structures. On a commercial scale, microbial products can be utilized in several ways such as:

- Direct application of fermentation produce in agriculture, medicine or any other sector.
- Products can be used as a starting material for derivatization process.
- The obtained compounds can act as leads in analogues synthesis and rational drug designing (Berdy, 2005[23]; Gupta et al., 2014[53]).

A favorable aspect of using microbes for the production of unique bioactive substances lies in the science of manipulating and improving microbial strains to achieve cost-effective production at industrial level by fermentation. Strategies for strain improvement involve two approaches:

- Classical genetic methods which include genetic recombination of microbes.
- Molecular genetic methods which include identification of biosynthetic pathways and effective transformation protocols (Gonzalez et al., 2003[50]).

#### Fungi

**Figure 9:**
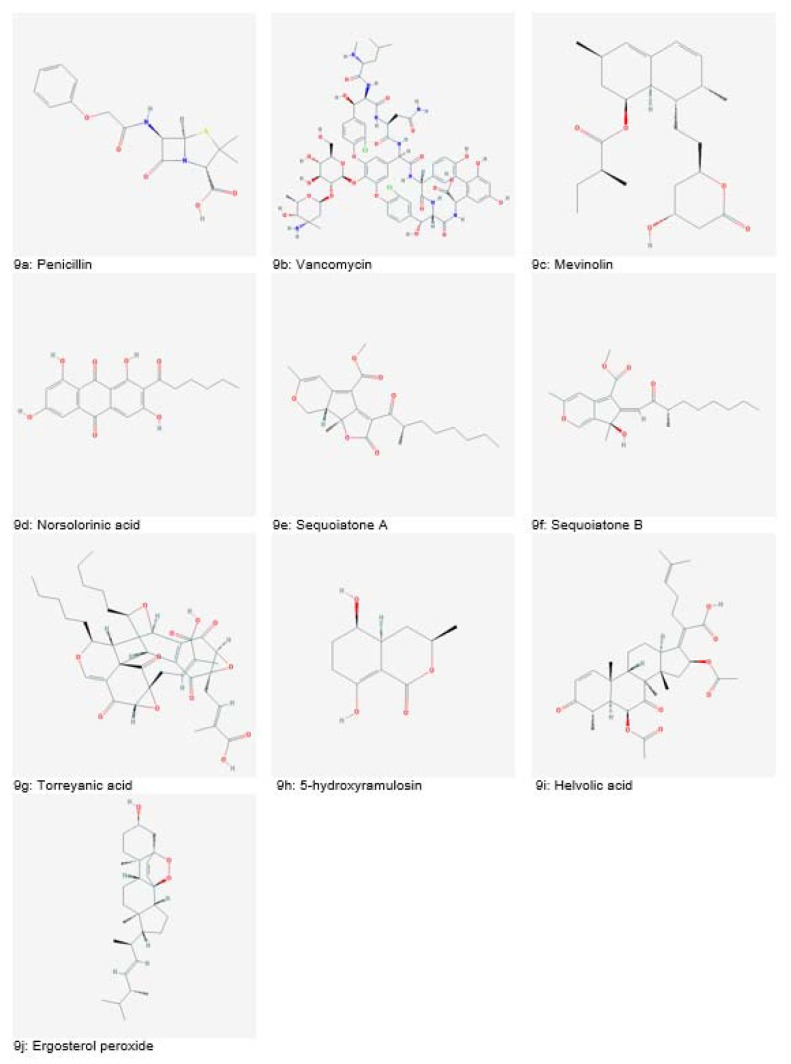
Natural bioactive compounds derived from fungi (Source: National Center for Biotechnology Information, PubChem Compound Database: CIDs from 9a-9j = 5904, 14969, 53232, 25102, 9978464, 15382209, 101697132, 54686464, 3002143 and 5351516)

The search for novel microbial metabolites began following the discovery of penicillin (Figure 9a(Fig. 9)) from *Penicillium notatum* in 1929 by Alexander Fleming. In 1953, another important discovery was the isolation of vancomycin (Figure 9b(Fig. 9)) an antimicrobial agent from *Amycolatopsis orientalis*. With the advancements in screening methods in the 1970s, several novel antibiotics such as norcardicin, imipenem and aztreonam were discovered (Dias et al., 2012[40]). 

Mevinolin (Figure 9c(Fig. 9)), a potent cholesterol-lowering agent was isolated from *Aspergillus terreus*. Aspercilin was isolated from *Aspergillus alliaceus*. Later on, benzodiazepines were derived from aspercilin and used for curing anxiety or insomnia (Gurnani et al., 2014[54]). Norsolorinic acid (Figure 9d(Fig. 9)), isolated from *Aspergillus spp. *was reported to cause apoptosis in breast cancer (MCF-7) and human bladder cancer (T-24) cells (Bladt et al., 2013[28]).

Extracts of *Penicillium steckii* and *Aspergillus sydowii* induced cytotoxicity in human cervical carcinoma cell line (HeLa) (Fajarningsih et al., 2013[42]). Whereas, extract of *Alternaria alternata *showed cytotoxic activity against *Staphylococcus aureus*, *Escherichia coli* and HeLa cells (Fernandes et al., 2009[44]). Similarly, Wu et al. (2014[180]) reported that ethanolic extracts of *Fomitopsis pinicola* induce cytotoxicity in various cancer cell lines including human hepatoma, colorectal, lung and breast cancer cells along with synergistic effects with cisplatin *in vivo*.

Many fungal species called endophytic fungi are inhabitants of intracellular spaces of plants without imposing harm. Various endophytic fungi have been investigated for bioactive compounds. One such example is *Cryptosporiopsis quercina*, isolated from *Tripterigeum wilfordii* (medicinal plant). It has shown potent antifungal activity against *Candida albicans* and *Trycophyton mentagrophyte.* Another endophytic fungus, *Aspergillus parasiticus*, isolated from *Sequoia sempervirens* was reported to be a producer of sequoiatones A and B (Figure 9e and 9f(Fig. 9)) which showed moderate anticancer potential with the highest activity against breast cancer cell lines. Torreyanic acid (Figure 9g(Fig. 9)), isolated from endophytic fungi of *Torreya taxifolia* tree exhibited apoptotic activity in protein kinase C sensitive cancer cells (Dias et al., 2012[40]; Gurnani et al., 2014[54]).

Endophytic fungi, *Taxomyces andreanae* and *Nodulisporium sylyiforme* have been reported to produce taxol (an anticancer drug previously isolated from the pacific yew tree). Along with anticancer potential, this drug also exhibited antifungal activity against *Pythium, Phytophthora and Aphanomyces spp*. (Gupta et al., 2014[53]). 

A novel polykedite, 5-hydroxyramulosin (Figure 9h(Fig. 9)) isolated from an endophytic fungus of *Cinnamomum mollissimum* showed both antifungal activities against *Aspergillus niger* and anticancer activity against murine leukemia cells (Santiago et al., 2012[166]). Helvolic acid (Figure 9i(Fig. 9)) and ergosterol peroxide (Figure 9j(Fig. 9)) obtained from endophyte *Pichia guilliermondii* inhabitant of *Paris polyphylla var. yunnanensis* plant have shown significantly strong antimicrobial activities against various bacterial pathogens (Zhao et al., 2010[186]). 

#### Bacteria

Natural habitats including soil, plant-associated environments, acidic water bodies, geothermal vents, high pH lakes, metal mining sites, extremely cold deserts, hot springs, marine soil sediments, radioactive waste disposal areas and many others are dwelling with a huge variety of microbes which are producers of structurally diverse compounds (Mahajan and Balachandran, 2014[73]). The extent of biodiversity among bacterial strains is much higher than that of plants and animals. For example, two bacterial strains, *Escherichia coli* and *Bacillus subtilis* are genetically more variable from each other than humans are from corns (Clardy, 2007[34]).

**Figure 10:**
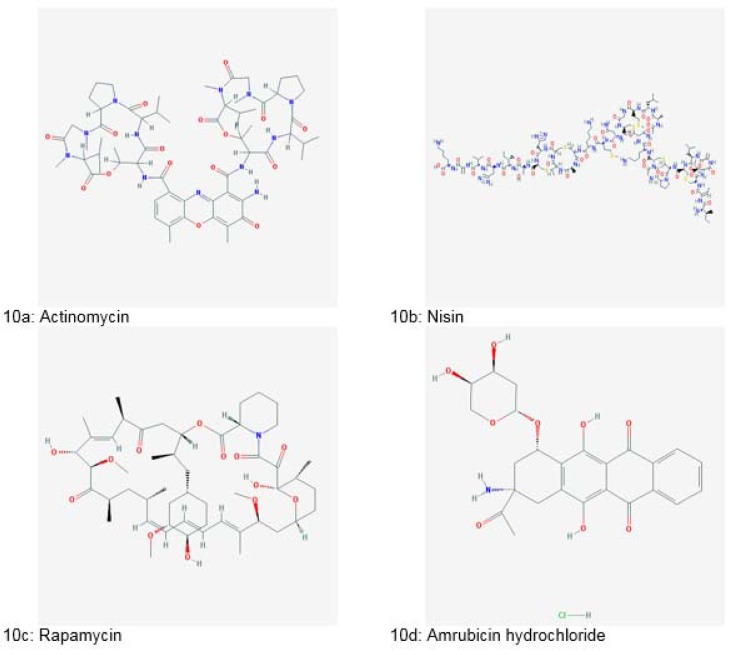
Natural bioactive compounds derived from bacteria (Source: National Center for Biotechnology Information, PubChem Compound Database: CIDs from 10a-10d = 2019, 16219761, 5284616 and 114897)

The search for bacterial metabolites in the field of therapeutics began after the discovery of an antibiotic, “Actinomycin” (Figure 10a(Fig. 10)) from gram-positive *Actinomycetes* in 1940. After that, many prominent anticancer drugs were isolated such as anthracyclines (daunorubicin, doxorubicin, epirubicin, pirarubicin and valrubicin), bleomycin, mitosanes (mitomycin C), anthracenones (mithramycin, streptozotocin and pentostatin), enediynes (calicheamicin), taxol and epothilones. Majority of them were isolated from *Streptomyces spp.* (Gupta et al., 2014[53]). 

Nisin (Figure 10b(Fig. 10)), a bacteriocin has been used as bio-preservative (Gupta et al., 2014[53]). Rapamycin (Figure 10c(Fig. 10)), an antifungal agent was isolated from soil inhabiting *Actinomycetes*. It is also used for inhibiting organ rejection in transplant patients (Clardy, 2007[34]). 

Amrubicin hydrochloride (Figure 10d(Fig. 10)), an anticancer compound was isolated from *Streptomyces peucetius* in 2002 (Dias et al., 2012[40]; Gurnani et al., 2014[54]). A new class of antibiotics called Pumalicidins A, B, C, D, E, F and G were obtained from the culture broth of *Bacillus pumilus*. Pumalicidin B resulted in 68 % reduction of Shay ulcers *in vivo *(Gupta et al., 2014[53]).

Homologues of Bacillomycin D, isolated from *Bacillus subtillis* (B38) were reported to have anti-oxidative and DNA protective activities (Singla et al., 2014[170]). Additionally, Surfactin, produced by *Bacillus subtilis* CYS191, was reported to induce apoptosis in human breast cancer cells (MCF-7) by causing oxidative stress (Cao et al., 2010[32]). *Streptomyces hygroscopicus* was identified as a producer of antifungal prenylated indole, galbonolides A and B, elaiophylin and its derivatives and herbimycins. Pterocidin, produced by endophytic *Streptomyces hygroscopicus* showed cytotoxic effects in human lung, ovarian, glioblastoma and melanoma cells (Igarashi et al., 2006[57]). Novel Methoxyneihumicin and Diketopiperazine isolated from *Nocardiopsis alba* exhibited anticancer activity (Singla et al., 2014[170]). 

Various strains of *Pseudomonas aeruginosa* have the ability to produce a copper-containing redox protein called azurin. Presence of copper gives azurin a number of properties which include blue colour and redox potential. Various domains of the protein are involved in anticancer, anti-parasitic and anti-HIV activities (Osman et al., 2013[152]). Punj et al*. *(2004[159]) reported that cytotoxic activity of azurin was largely dependent on the presence of p53 gene. Similarly, an analogue of signal peptide 27, produced by *Streptococcus pneumoniae* has shown cytotoxicity against leukemia, gastric and breast cancer by cellular permeabilization and induction of caspase-independent apoptosis. Another anticancer peptide Entap (Enterococcal anti-proliferative peptide) isolated from *Enterococcus spp*. cause induction of autophagous apoptosis and inhibits proliferation in several cancers (Karpinski and Szkaradkiewicz, 2013[61]).

Recently, bacterial toxins are gaining interest in targeted cancer therapy (Table 3(Tab. 3); References in Table 3: Foss, 2000[46]; Karpinski and Szkaradkiewicz, 2013[61]; Nair at al., 2014[80]; Pahle et al., 2017[153]; Patyar et al., 2010[155]; Yang et al., 2013[181]; Zahaf and Schmidt, 2017[183]). They are produced naturally as virulence factors by various strains of pathogenic bacteria. High stability and immunogenicity make them well-suited candidates for cancer therapy. Bacterial toxins are now being used as immunotoxins which contain a toxin moiety attached to a target-specific antibody (Becker and Benhar, 2012[22]).

## Highlights and Challenges of Natural Drug Discovery

Drug discovery from natural resources has its own pros and cons. Some of them are mentioned below:

### Highlights

- Natural compounds have a great amount of biodiversity.
- They are bioactive in nature because they are produced as a result of constant evolution among organisms.
- They are supported by folklore as well which accounts for more public acceptance.

### Challenges

- Working with crude extracts, their fractions and assessing their pharmacological potential is difficult.
- The concentration of a bioactive entity in an extract or fraction is uncertain.
- Optimizing the culture conditions for maximum production of the desired compound can be challenging especially in case of microbes.
- Enzyme-based screening tests may be difficult to perform due to biological interactions with natural products.
- Natural products possess complex structures which make them tough to synthesize.
- Bioassays need to be conducted at every step of product isolation, making the process time-consuming.
- Isolation of unstable compounds, separation difficulties and use of unreliable bioassays may also make the process of drug discovery slow.
- Re-isolation of previously known compounds can result in resources insufficiency.
- Not all natural compounds are favourable drug leads. They may require structural modifications.
- New strategies may be required to further exploit already screened biological resources (Beutler, 2009[24]; Siddiqui et al., 2014[168]).

## Modern Approaches in Natural Product Research

The process of natural drug discovery has been divided into two approaches:

- Top-down approach
- Bottom-up approach

### Top-down approach

Top-down approach has been used traditionally for the discovery of natural bioactive compounds. It includes extensive sampling from diverse habitats, from which extracts are prepared for screening purpose. After observing the desired bioactivity, structural characterization of active compound is done. The top-down approach is organism-based which does not involve previous knowledge of genetic makeup of enzymes of a producer. It is more suitable for compounds which are reproducibly produced in native environment or laboratory. With the advancements in screening techniques i.e., the introduction of high throughput screening methods, sensitivity towards detection of small novel compounds and compounds present in limiting amounts have been increased (Luo et al., 2014[72]). 

### Bottom-up approach

The bottom-up approach is based on the identification of gene clusters involved in the synthesis of desired bioactive compound and use of genetic manipulation techniques to promote its synthesis. Genomic-based approach has helped in identification of novel lead compounds which were not detectable under normal fermentation techniques. It includes the use of bioinformatics tools, functional genomics and gene manipulation approaches (Luo et al., 2014[72]).

- Bioinformatics tools such as SMURF (Secondary Metabolite Unknown Regions Finder) and anti SMASH (Antibiotics & Secondary Metabolite Analysis Shell) are helpful in identification of specific gene clusters responsible for the manufacturing of bioactive substances. SMURF was the first tool to identify gene clusters for polyketides, terpenes, alkaloids, non-ribosomal peptides and polyketides in fungal genome. However, anti SMASH is capable of identifying gene clusters for known natural compound classes from any genomic sequence (Luo et al., 2014[72]; Medema et al., 2011[76]).
- With the advancement in gene knockout technology and metabolomic profiling, it has become feasible to distinguish gene clusters responsible for producing a specific product or to discover unknown or previously undetected products in native hosts. For example, cichorine (phytotoxin) producing gene clusters were identified in *A. nidulans*. Gene deletion techniques have also been implied to activate silent gene clusters in organisms. An example is echinocandin B producing gene cluster in *Emericella rugulosa* NRRL 11440. Another deletion based technique to activate otherwise silent gene clusters involves knockout of kinases which are key regulators of signal transduction (Sanchez et al., 2012[165]; Cacho et al., 2012[31]).
- Gene knock-in technology has also shown great potential to activate or enhance the expression of silent gene clusters involved in the synthesis of secondary metabolites. Non-reducing polyketide synthase (NR-PKS) gene cluster involved in the synthesis of natural products has been targeted to enhance the production. In *Burkholderia thailandensis*, insertion of a constitutive promoter in front of burA gene of nonribosomal peptide synthetase (NRPS)-PKS cluster has resulted in the discovery of polyketide burkholderic acid (Liu and Chang, 2014[71]; Luo et al., 2014[72]).
- Heterologous gene expression offers an advantage as genetic manipulation in native hosts is difficult or not well developed in many organisms. It involves the expression of a gene of interest, gene cluster or gene cassette in a suitable host to identify the natural product. For example, *Saccharomyces cerevisiae*, has been utilized as a host for the expression of PKS and NRPS genes isolated from fungi. Similarly, *E. coli* has been utilized in heterologous expression of terpene cyclases where post-translational modification is not required and only one fungal enzyme is needed to be expressed. Cryptic pathways have been activated in heterologous hosts by introducing strong promoters into the gene clusters with the help of RecET direct cloning technique (Alberti et al., 2017[12]; Luo et al., 2014[72]).
- Other techniques involve the use of DNA assembler which aids in the reconstruction of gene clusters resulting in the production of novel entities. This combinatorial biosynthesis strategy has been applied successfully in the production of novel erythromycin analogs using *E. coli* as heterologous host (Zhang et al., 2015[185]).

## Latest Advancements in Natural Product Discovery

- Future holds great potential for natural product discovery due to the establishment of genomic profiles and other bottom-up approaches.
- In order to discover novel bioactive entities with adequate pharmacokinetics, bioavailability and efficacy from natural sources, a combination of different approaches i.e., bottom-up, top-down, synthetic library screening, combinatorial synthesis, structure-based designing must be used.
- Introduction of SepBox technique which is a patented combination of high-performance liquid chromatography (HPLC) and solid-phase extraction (SPE) and is providing an opportunity for automated isolation of compounds from extracts or fractions.
- With the advancements in nuclear magnetic resonance imaging (NMR) techniques such as cryogenic NMR, capillary NMR, and LC-NMR, it is now possible to screen ligand and protein interactions.
- Use of de-replication process to distinguish known bioactive compounds in extracts before doing bioassay based isolation has provided aid in reducing research time and selection of important samples for further analysis.
- Metabolomics advances will allow foretelling chemical composition of extracts with the help of transcriptome, proteome and genome data (Bhandari et al., 2011[25]; Gurnani et al., 2014[54]; Rye et al., 2017[163]; Siddiqui et al., 2014[168]).

## Conclusions

Natural substances are the foundations of novel therapeutic lead compounds with minimal side effects. This is because of the presence of tremendous biodiversity among plants, animals, marine organisms and microorganisms. The process of drug discovery from natural sources is slow and monotonous and is associated with uncertain results. However, with the help of recent advancements such as proteomics, genomics, transcriptomics and genetic modification, several natural products can now be screened for their bioactivity which may contribute towards future drug development.

## Conflict of interest

The authors declare no conflict of interest.

## Figures and Tables

**Table 1:**
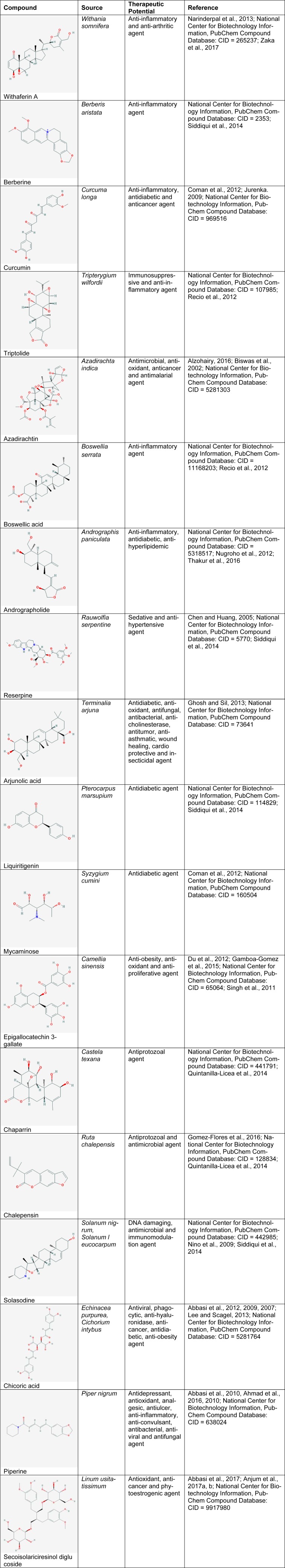
Important plant-based bioactive entities

**Table 2:**
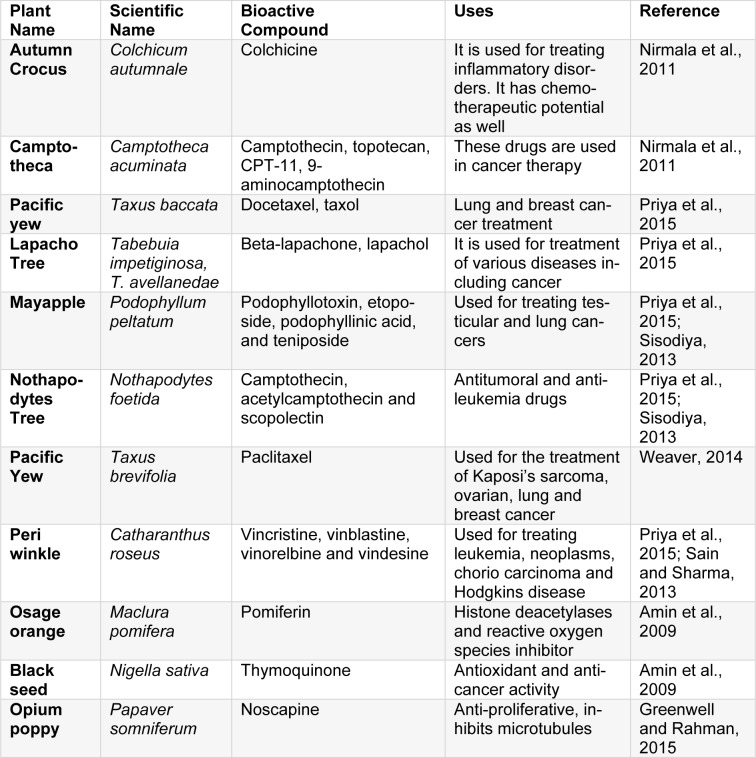
Important anticancer medicinal plants

**Table 3:**
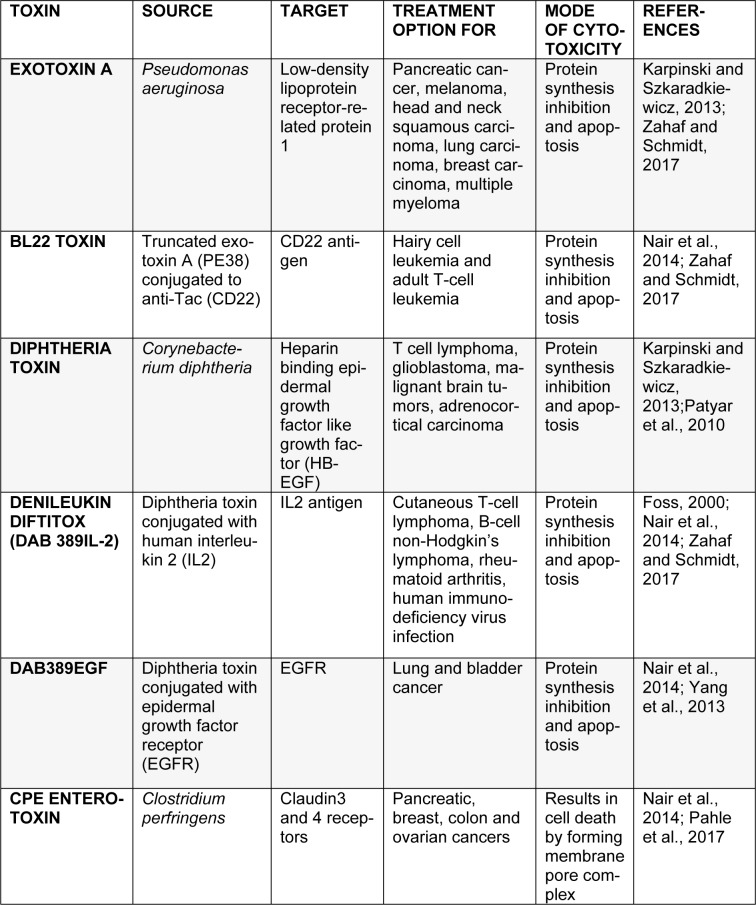
Bacterial toxins and immunotoxins, their sources, targets and mode of action
